# Can different exercise modalities improve esports performance? Study protocol for a 12-week three-arm randomized controlled trial

**DOI:** 10.1371/journal.pone.0357119

**Published:** 2026-08-25

**Authors:** Di Tang, Zheng Ye, Ruisi Ma, Jinde Liu, Wai Keung Ho, Raymond Kim Wai Sum

**Affiliations:** 1 Department of Sports Science and Physical Education, Faculty of Education, The Chinese University of Hong Kong, Hong Kong, China; 2 The Nethersole School of Nursing, Faculty of Medicine, The Chinese University of Hong Kong, Hong Kong, China; 3 School of Physical Education, Jinan University, Guangzhou, China; 4 Faculty of Physical Education, Fudan University, Shanghai, China; İzmir Democracy University: Izmir Demokrasi Universitesi, TÜRKIYE

## Abstract

**Background:**

Growing evidence indicates a positive association between physical exercise and both cognitive functions and gaming performance in esports players. However, most existing studies focus on acute or short-term effects and are limited by small sample sizes and a lack of follow-up assessments. Furthermore, comparisons between different exercise modalities remain largely unexplored. Therefore, longitudinal studies comparing optimal exercise protocols and their sustained effects on esports performance are critically needed.

**Objective:**

This study aims to: (1) investigate and compare the long-term effects of 12-week high-intensity interval training (HIIT) and moderate-intensity continuous training (MICT) on various dimensions of esports performance in first-person shooter (FPS) gamers; (2) examine the sustainability of these effects through a 4-week follow-up assessment; and (3) explore the potential associations between physical competence, psychological skills, and gaming performance.

**Methods:**

In this three-arm randomized controlled trial, 195 FPS gamers (aged 18–29) will be randomly assigned to HIIT, MICT, or control groups. Interventions consist of three weekly sessions for 12 weeks (HIIT: 10 × 1-minute high-intensity bouts interspersed with 1-minute active recovery; MICT: 20-minute continuous moderate-intensity exercise). The primary outcome is flicking capacity (a core metric of esports performance). Secondary outcomes include other gaming operational metrics, esports-related psychological skills, body composition, and physical competence. All outcomes will be assessed at baseline, post-intervention (12 weeks), and follow-up (16 weeks).

**Discussion:**

This protocol outlines a standardized longitudinal framework to address the critical lack of evidence-based esports training guidelines. Anticipated findings will further validate the benefits of physical exercise on esports performance, providing essential long-term empirical evidence for integrating optimal exercise modalities into esports training regimens. Furthermore, exploring the associations between physical competence, psychological skills, and operational performance will offer insights into the behavioral pathways of performance enhancement.

**Trial registration:**

ClinicalTrials.gov (NCT07150312). Registered on Aug 29, 2025**.**

## Introduction

Despite its growing prominence, esports lacks a universally standardized definition [1,2], though it is widely characterized as competitive sports facilitated by electronic systems and human-computer interfaces [1,3]. While the industry has achieved substantial global growth [3–6] and mainstream recognition as an official sport [7–9], evidenced by its inclusion in the Asian Games and acknowledgment by the International Olympic Committee, systematic research on esports training and performance lags significantly behind traditional sports [10], highlighting a critical need for scientific evidence to support sustainable competitive development [11–13].

Research in neuroscience, psychology, and sports science suggests that aerobic exercise may enhance cognitive functions relevant to gaming performance, with particular effects on executive control processes. These processes, which include information filtering and rapid decision-making [14–16], are actively involved in video gaming [17,18]. Furthermore, regular physical exercise has been shown to improve stress management and anxiety control, while also enhancing reaction ability and communication skills [19–21], all of which are fundamental components for maintaining optimal performance in competitive esports settings [22,23]. Supporting this connection, studies have identified correlations between cardiorespiratory fitness and reaction time among esports players [24].

Emerging empirical evidence has begun to substantiate the theoretical link between physical activity and esports performance [25–33]. For instance, brief active breaks, such as six-minute walks during gaming sessions, have been documented to enhance executive function in esports players [26]. Additionally, multiple studies have demonstrated the positive effects of pre-game cardiovascular workouts on performance metrics, collectively validating the immediate benefits of physical exercise on esports performance [27,28,34,35].

Nevertheless, current research presents several methodological limitations that warrant attention, including insufficient sample sizes that limit statistical power, predominantly immediate or short-term interventions focusing on acute effects, a lack of follow-up assessments to evaluate the sustainability of effects, and an absence of comparative analyses across different exercise modalities [12,29]. As a training modality with substantial potential for enhancing competitive performance, understanding the long-term adaptations and sustained effects of physical exercise is crucial for developing evidence-based training protocols in esports [12,27,32].

In previous intervention studies, two exercise modalities that have shown potential benefits for improving cognitive function and esports performance are high-intensity interval training (HIIT) and moderate-intensity continuous training (MICT). HIIT involves short bursts of intense exercise followed by rest or low-intensity exercise [36]. This training approach has been associated with significant improvements in cardiorespiratory fitness and has demonstrated unique neurocognitive benefits [37]. Additionally, a single bout of HIIT has been shown to improve esports performance in multiplayer online battle arena (MOBA) gamers [27]. In contrast, MICT involves steady-state aerobic activity maintained at 40–60% of maximum heart rate for extended periods. This approach also has well-established benefits for cognitive function, particularly in domains of sustained attention, working memory, and executive function [38–41]. The mechanisms underlying MICT’s cognitive benefits appear to differ from those of HIIT, involving more gradual but sustained increases in cerebral perfusion, reduced inflammation, and enhanced vagal tone [42–44]. These distinct physiological adaptations suggest that MICT might benefit different aspects of esports performance compared to HIIT, particularly in gaming scenarios requiring prolonged concentration and performance consistency. The differential effects of HIIT versus MICT on brain function have been demonstrated in several studies [45–49]. For example, research shows that while both exercise types can improve cognitive functions, they often do so through different mechanisms and with varying degrees of effectiveness. HIIT tends to show stronger effects on processing speed, while MICT may better enhance sustained attention [48–50]. However, while these physiological and psychological differences between exercise types have been studied in general populations, no research has directly compared these modalities in esports players or measured their effects on actual gaming performance in long-term interventions.

First-person shooters (FPS) hold a prominent position within the esports landscape. These games require players to demonstrate exceptional hand-eye coordination, reaction time, and decision-making skills [51]. Moreover, shooters may be more demanding of players’ reaction time than other esports games, and overall performance is heavily dependent on players’ reaction levels, with reaction time being one of the most essential abilities in FPS [24,51]. These games demand both quick reflexes for moment-to-moment engagements and sustained strategic awareness throughout matches. This combination of performance requirements makes FPS particularly suitable for comparing how different exercise modalities might enhance distinct aspects of gaming performance.

Currently, a standardized or universally agreed-upon method for gauging esports performance has yet to be established. Within the esports arena, it is recognized that performance encompasses not merely physical attributes such as stability of the upper limbs, eye-hand coordination, and manual agility, but also cognitive and psychological dimensions, including resilience to stress, responsiveness, problem-solving skills, and the ability to multitask [22,52]. Existing research predominantly utilizes three measurement approaches: rank-based evaluations [12,53], which provide a holistic view but can be influenced by other confounding factors beyond performance; customized performance tasks simulating specific in-game operations [27,34,54,55], which offer direct insights but often fail to replicate complex competitive environments, thus falling short of assessing holistic, multifaceted performance; and indirect psychological and physiological assessments [26,56,57], which are scientifically robust but may not accurately represent in-game performance [58]. Each method has its strengths and limitations in comprehensively evaluating esports skills, highlighting the need for a more integrated approach to assessment.

Building on our previous 6-week study, which showed no significant improvements in psychological attributes among esports players, we hypothesize that these null findings may stem from insufficient exercise intensity and duration [32]. Given that existing literature consistently identifies psychological skills as fundamental determinants of competitive esports success [12,22,59,60], verifying whether an optimized exercise regimen can effectively enhance these attributes remains an objective of our research. To address the aforementioned limitations in current exercise-esports research and to further advance our previous findings, the current study employs a three-arm randomized controlled trial to compare the effects of a 12-week HIIT and MICT intervention on esports performance and psychological skills, followed by a 4-week follow-up to evaluate the sustainability of these effects. By analyzing the relationships between these performance indicators and physical fitness indicators, we aim to explore the potential underlying mechanisms of different exercise modalities. Ultimately, the findings will offer evidence-based guidance for esports organizations, advancing our understanding of the exercise-psychology-performance relationship in competitive gaming.

## Participants and methods

**Fig 1:**
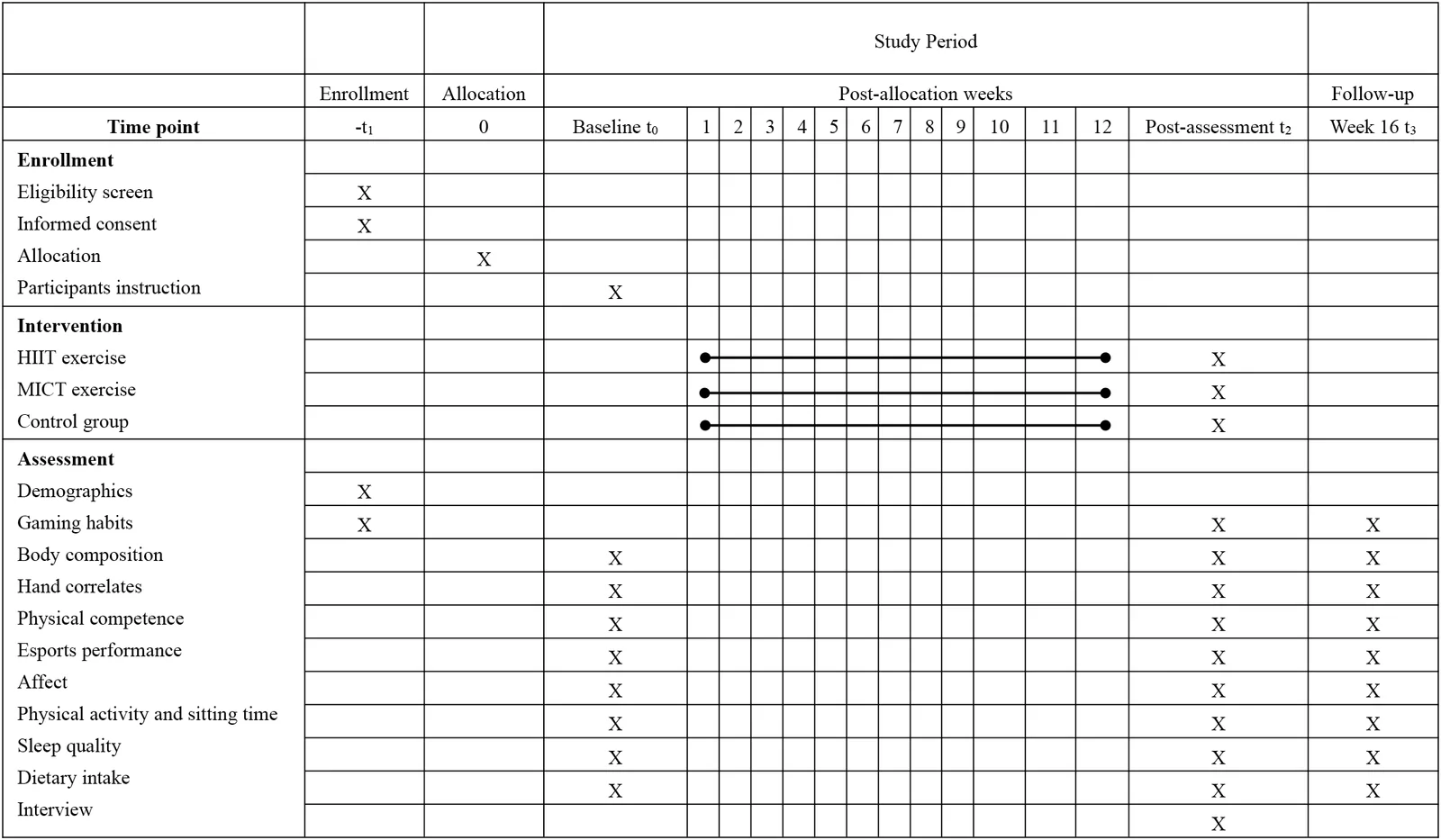
Schedule of enrollment, interventions, and assessments. Note: HIIT: High-Intensity Interval Training; MICT: Moderate-Intensity Continuous Training. -t_1_:pre-allocation/enrollment; t_0_: baseline assessment; t_2_: post-intervention assessment (week 12); t_3_: follow-up assessment (week 16). The symbol “X” indicates that a specific assessment or procedure is performed at that time point. The solid line with dots indicates the continuous 12-week intervention period. For detailed descriptions of the specific instruments and tools used for each assessment variable, please refer to the “Measures” section in the main text.

This research follows the SPIRIT (Standard Protocol Items: Recommendations for Interventional Trials) guidelines (see S1 Checklist), detailing both our current intervention approach and the randomized controlled trial protocol. The entire study is anticipated to take between 18 months to 24 months to complete. Fig 1 outlines the approximate schedule for the study and the estimated duration.

### Trial registration

The protocol has been registered with ClinicalTrials.gov. The registration number is NCT07150312, and the registration date is 29-08-2025.

### Research objective

This study aims to investigate the long-term effects of HIIT and MICT on esports performance through a 12-week three-arm randomized controlled trial, and to examine the sustainability of these effects with a follow-up assessment conducted 4 weeks after the intervention cessation. Additionally, we seek to compare the differential impacts of these two exercise modalities on various dimensions of esports performance and to explore their associations with changes in body composition and physical competence, thereby providing evidence-based guidance for esports training regimens and enhancing our understanding of the underlying mechanisms by which different exercise approaches influence esports performance.

### Study setting

Measurements and initial parts of the supervised exercise intervention in this study will be conducted in the laboratory of the Department of Sports Science and Physical Education at The Chinese University of Hong Kong. The latter stages of the exercise intervention will be carried out by the participants at their own homes.

### Participants

We will recruit healthy adult FPS gamers of any gender for this study through the university’s mass mail websites and social media platforms such as WeChat groups, and Instagram. Additionally, the QR code for the initial online survey will be displayed at offline esports activities for anyone interested in participating. The age range of participants will be set between 18 and 29 years for two reasons: firstly, this age range represents the primary demographic of current esports participants and professional esports athletes [61,62]; Secondly, setting the age limit at 18 is also in consideration of the regulations of the Chinese National Radio and Television Administration regarding the participation of minors in video games. Under the regulations of the National Radio and Television Administration and the implementation of the Chinese Online Game Fatigue System in games, minors have limited opportunities to engage in esports gaming [63]. Therefore, in compliance with national regulations and for the protection of minors, participants under the age of 18 will not be recruited in this study. As for the determination of esports players, based on previous literature, we will recruit players who have engaged in esports games within the past six months [32,62,64]. In summary, our inclusion and exclusion criteria are as follows:

Inclusion criteria:

- 18–29 years old;
- regular FPS players with at least 1 year of FPS gaming experience, who have played FPS games for a minimum of 5 hours per week over the past six months [27];
- healthy individuals who are capable of physical exercise and tests, assessed and confirmed
  by the Physical Activity Readiness Questionnaire (PAR-Q) [65];
- no regular exercise habits (engaging in < 2 structured exercise sessions per week) [66,67];
- can understand English or Chinese.

Exclusion criteria:

- self-reported history of neurological, psychiatric, or medical diseases;
- having heart disease, hypertension, musculoskeletal injuries, or any other conditions that pose a risk to engaging in physical activity
- current intake of medications and/or recreational drugs that could affect the central nervous system and/or the ability to learn;
- a regular user of Aim Lab (used Aim Lab more than once a week in the past six weeks), to prevent baseline bias and potential learning effects, as Aim Lab will be utilized as the primary training and assessment tool in our subsequent intervention.

During the recruitment, we will conduct a preliminary screening of potential candidates through an online questionnaire survey based on our inclusion and exclusion criteria.

### Experimental design

**Fig 2:**
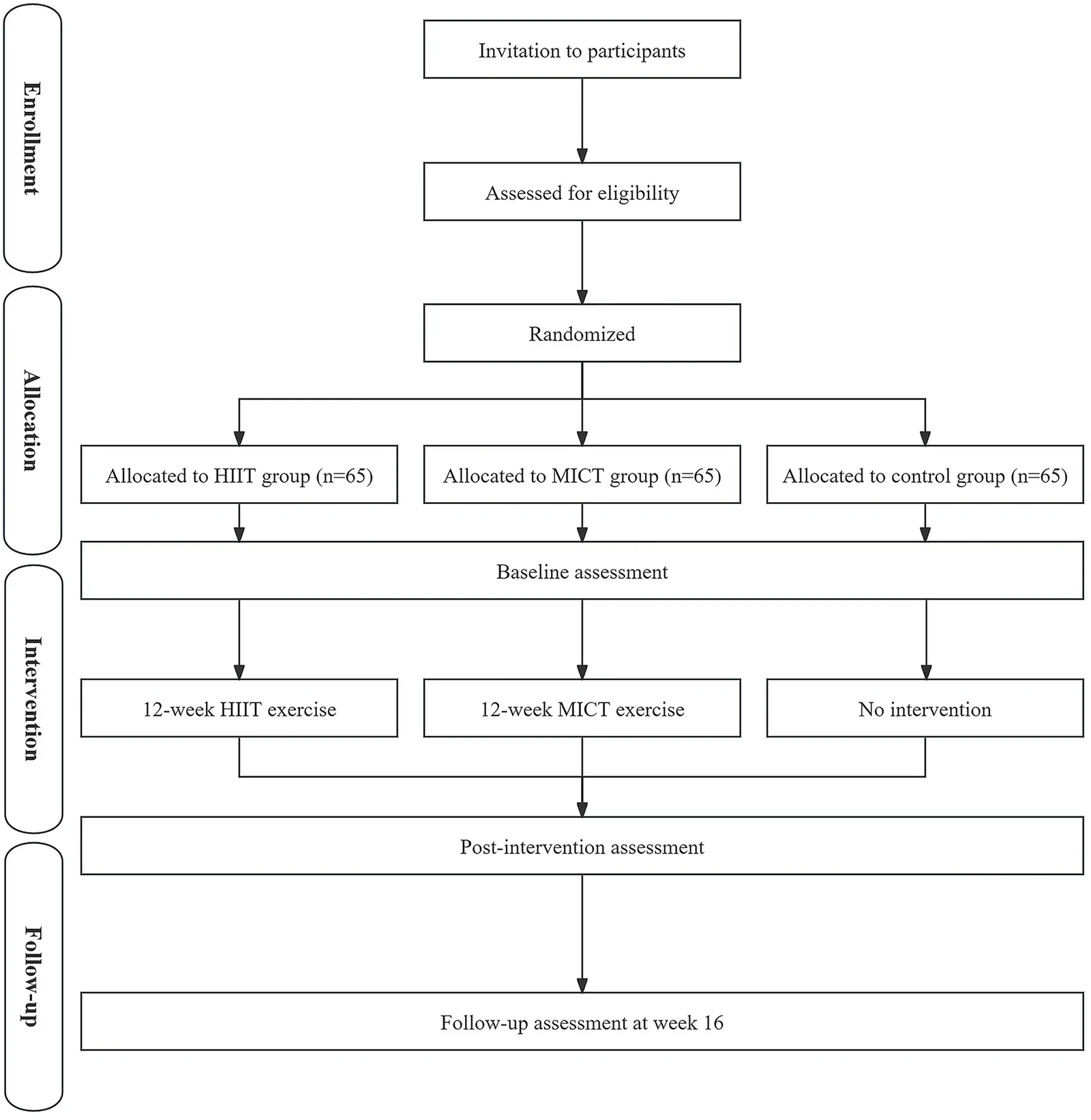
Flow chart of the experimental procedure.

This study is designed as a three-arm randomized controlled trial. Given the limited number of longitudinal studies examining exercise interventions in esports, our sample size estimation was informed by the results of existing acute or short-term exercise studies aimed at enhancing esports performance, alongside preliminary data from our previous research [27,35]. Based on the effects of exercise on esports performance reported in these studies, we conservatively estimated a moderate effect size (d = 0.4) to power our primary outcomes (esports performance). Power analysis conducted via G*Power indicated that a minimum sample of 52 participants per group would be required to detect significant effects with 80% power (α = 0.05). Accounting for an anticipated dropout rate of 20%, 65 participants for each group will be recruited for this study. In total, we will recruit 195 participants, and they will be randomly allocated into three groups (1:1:1): HIIT, MICT, and a control group. To prevent baseline imbalances in variables relevant to gaming performance, a stratified block randomization procedure will be employed. Participants will be stratified into two strata based on their years of FPS gaming experience (e.g., above vs. below the median years of experience of the recruited cohort). Within each stratum, participants will be randomly allocated to the HIIT, MICT, or control group in a 1:1:1 ratio using randomly permuted blocks. The randomization sequence will be generated by an independent researcher using a computer-based random number generator (Research Randomizer) to ensure allocation concealment. The entire allocation process will be conducted by a staff member who will not be participating in the study. In scenarios requiring assessors to utilize measurement tools for evaluating metrics, we engage testers who are completely blind to the participants’ group assignments to conduct the assessments. For metrics tested via computer, our assessors serve merely to facilitate and oversee the process, with the computer’s saved data serving as the definitive record to ensure data objectivity. This approach has been proven reasonable in the past esports performance assessment [27]. The intervention groups will receive a 12-week HIIT or MICT exercise program, while the control group will not receive any intervention. All groups will report their dietary intake, physical activity, sleep patterns, and gaming activity. All participants will undergo a baseline test, followed by an immediate post-test at the end of the 12-week intervention period and a follow-up test 4 weeks after the intervention ends. Fig 2 illustrates the specific experimental procedure.

### Measurements

To comprehensively evaluate the effects of the 12-week exercise intervention, the measurements in this study are systematically categorized into primary outcomes, secondary outcomes, exploratory outcomes, and control and monitoring variables. The core construct of our investigation is esports performance, which we conceptualize as a multidimensional framework comprising both objective operational skills and esports-related psychological skills. To implement our multidimensional evaluation of esports performance while maintaining strict statistical rigor, the primary outcome is specifically defined as flicking capacity in esports performance (quantified by the total number of targets successfully hit during a 1-minute task). This metric was strategically selected as the primary endpoint because it effectively captures “aiming throughput”, inherently accounting for the speed-accuracy trade-off by rewarding both speed and precision.

The secondary outcomes encompass the remaining dimensions of our esports performance framework, including other operational metrics (detection capacity, tracking performance, overall shooting accuracy, and reaction speed) and esports-related psychological skills, as well as body composition and physical competence.

Other outcomes were strategically selected to support and enhance the validity of our primary analysis while maintaining a clear focus on the main research objectives. These measures serve three specific purposes: (1) to identify and control for potential confounding variables through covariate analyses, ensuring robust assessment of the exercise’s effect on esports performance; (2) to monitor and standardize key lifestyle factors (e.g., sleep, affect, physical activity, and dietary intake) known to influence cognitive and gaming performance, thereby isolating the intervention effect; and (3) to enable supplementary analyses exploring potential mechanisms underlying the relationship between physical exercise and esports performance.

#### Esports performance.

To comprehensively evaluate the objective operational skills, we utilized a specialized FPS training software called Aim Lab. Aim Lab is a free PC application designed to enhance aiming performance in FPS games. It offers comprehensive testing, training, and analysis features tailored to assess and improve players’ aiming abilities. Aim Lab has been employed in research on esports performance within the FPS gaming domain [68,69]. We selected five types of skill tests that are highly relevant to shooting games: flicking, tracking, accuracy, detection, and speed. Specifically, flicking capacity and detection capacity will be quantified by the total number of targets successfully hit during their respective 1-minute tasks. Tracking performance will be measured by the total time (in seconds) the participant successfully keeps the crosshair locked onto a moving target during the 1-minute tracking task. Furthermore, overall shooting accuracy and reaction speed will be evaluated. Accuracy will be calculated as the average hit rate (the percentage of successful hits out of total shots fired), while speed will be determined by the average reaction time (in milliseconds), with both metrics derived from the combined data of the flicking and detection tasks.

Choosing to use a third-party shooting program for testing instead of a specific game's custom approach has several advantages. Firstly, it ensures that all shooting game players have the same level of familiarity with the testing program, preventing individual players from obtaining higher esports performance scores due to their proficiency in a chosen game. Secondly, Aim Lab is primarily a training program focused on practice rather than entertainment, and it is unlikely that casual players would engage in regular training with this program. By selecting this software, we can avoid the potential influence of players’ habitual use of Aim Lab during the 12-week intervention, which could enhance their proficiency and subsequently affect the final performance test. Additionally, we excluded habitual users of Aim Lab during the screening phase to mitigate this issue. On the other hand, using a specific mainstream shooting game for testing would make it difficult to control the spontaneous participation of players who are loyal users of those games during the 12-week intervention, leading to an increase in proficiency and potentially affecting the final game performance test. Furthermore, Aim Lab, being a dedicated shooting training and testing software, provides detailed test scores for different dimensions of shooting, such as reaction time and accuracy. Compared to previous studies that used custom modes within specific esports games for testing, using Aim Lab offers a more objective approach, avoiding subjective factors or errors dependent on assessors [27].

Before the Aim Lab test begins, each participant will undergo a warm-up session to familiarize themselves with the software, mouse, and keyboard setups. Razer Synapse will be used to help participants set the DPI (Dots per inch). All Aim Lab tests will be conducted on the same computer in our laboratory, using identical mouse and keyboard setups.

Considering the enhancement of psychological qualities in participants through physical exercise, as well as the correlation between esports performance and psychological skills, we have implemented three types of psychological tests in addition to the operational gaming tests. These tests include psychological skill use, self-regulation, and mental toughness. These psychological measures, specifically psychological skill use, self-regulation, and mental toughness, have been demonstrated in previous research to have significant correlations with gaming performance and competitive abilities in esports players [59,60]. The Self-Regulation Questionnaire (SRQ) [70,71], Test of Performance Strategies-2 (TOPS-2) [72], and Sports Mental Toughness (SMTQ) [73] will be used, and these qualities were proven to be correlated with esports performance [59,74]. To ensure accurate comprehension by the participants, bilingual (English-Chinese) versions of these instruments were administered. The Chinese translations of these scales have been previously validated and have demonstrated robust psychometric properties, including excellent internal consistency and structural validity, within Chinese populations [75–77]. SRQ is composed of 63 items divided into six distinct dimensions: evaluating, triggering, searching, planning, implementing, and assessing. Responses to the SRQ are measured using a 5-point Likert scale. TOPS-2 features a total of 64 questions, split evenly, with 32 focused on competitive scenarios and another 32 pertinent to practice settings. For the purposes of our research, we exclusively considered the subset of questions that pertain to competitive contexts. The TOPS-2 encompasses a range of nine psychological dimensions that are believed to influence performance. These include the establishment of objectives, the use of mental imagery, techniques for achieving calmness, methods of energy management, internal dialogue practices, management of emotions, the development of automated responses, patterns of pessimistic thought, and susceptibility to distraction. However, in this investigation, the susceptibility to distraction was omitted from the analysis based on prior studies that have indicated its lower reliability in terms of internal consistency [72]. The SMTQ is a 14-item instrument that was established to ascertain athletes’ mental toughness levels. SMTQ is structured into three distinct subsections: it includes a set of six questions aimed at assessing confidence, another four that measure steadiness, and an additional four items dedicated to evaluating an individual’s level of self-regulation.

#### Body composition and anthropometry.

Body composition will be assessed using a multi-frequency bioelectrical impedance analyzer (MC-780MA, Tanita Corporation, Tokyo, Japan). Key parameters extracted will include body weight, body mass index (BMI), fat mass and percentage, as well as total and regional (upper and lower limb) muscle mass.

Additionally, specific anthropometric data will be recorded. Waist and hip circumferences will be measured using a standard non-elastic tape. Furthermore, considering the specific operational demands of esports, participants’ hand dimensions, including finger length, palm length, and palm width, will be precisely measured. These hand anthropometrics are collected to account for physical traits that may potentially influence mouse-grip mechanics and gaming operational performance.

#### Physical competence.

In this domain, six variables will be measured using well-established and standardized field tests. These include maximal oxygen consumption (VO2max), grip strength, core strength, body balance, agility, and flexibility. VO2max will be assessed through PACER (Progressive Aerobic Cardiovascular Endurance Run) 20-m [78,79]. Grip strength will be measured with a handgrip dynamometer [80]. The core strength, static balance, agility, and flexibility will be measured, respectively, through the Plank Test [81], Unipedal Stance Test [82], Agility T-Test [83], and Shoulder Reach Test [84].

To prevent mutual impact, the test of physical competence will be conducted on a different day from the assessments of esports performance. There will be a minimum 48-hour gap between these two sets of assessments.

#### Perception.

Beyond the aforementioned quantitative metrics, we will conduct supplementary, semi-structured interviews following the completion of the 12-week intervention. We will invite all participants from the two exercise intervention groups (HIIT and MICT) who consent to engage in subsequent dialogues. Because this assessment is strictly tied to the intervention, the sample size is predetermined by the number of intervention completers. Consequently, participant recruitment is not dictated by traditional qualitative data saturation criteria. The interviews will be highly open-ended, with the primary guiding question being: “How do you perceive the 12-week exercise intervention has affected your in-game esports performance?”. Follow-up probing questions will be used to encourage participants to elaborate on specific subjective feelings, such as perceived changes in focus, fatigue, or operational mechanics. The impetus for this supplementary qualitative method is twofold: firstly, to explore participants’ subjective expectations to evaluate potential expectancy effects stemming from the lack of participant blinding; and secondly, to capture subjective experiences that can offer valuable directional insights and hypotheses for future research in this under-investigated field.

#### Affect.

Before the esports performance assessment, participants are required to fill out the bilingual (English-Chinese) version of the International Positive and Negative Affect Schedule Short Form (I-PANAS-SF) [85]. This scale was used to determine whether short-term exercise-induced changes in affect influenced video game performance. The I-PANAS-SF is a brief, self-reported questionnaire that consists of two 5-item scales (10 items in total) to measure both positive and negative affect. The Chinese version of the I-PANAS-SF has also been validated, demonstrating excellent internal consistency and cross-cultural measurement invariance within Chinese populations [86].

#### Physical activity and sitting time.

The evaluation of both physical activity and sitting time will be conducted through the bilingual (English-Chinese) version of the self-administered International Physical Activity Questionnaire (IPAQ) long form. This is a widely used instrument, and its Chinese translation has well-established reliability and validity among Chinese cohorts [87,88]. This specific version of the IPAQ comprises a set of 27 questions designed to ascertain the duration (in minutes) of participants’ involvement in physical activities of different intensity levels.

Moreover, to prevent the inclusion of participants who already had a regular habit of engaging in aerobic exercise, spontaneously engaging in additional aerobic exercise during the 12-week intervention period that could potentially influence the final test results, we excluded players who already had a regular habit of engaging in cardiovascular exercise during the recruitment process.

#### Sleep quality.

Participants will be asked to maintain their usual sleep habits over 12 weeks, and they are required to complete the bilingual (English-Chinese) version of the Pittsburgh Sleep Quality Index (PSQI) [89] both before and after the pre-test and post-test sessions. The PSQI is a 19-item self-rated questionnaire for evaluating subjective sleep quality over the previous month. The 19 questions are combined into 7 clinically derived component scores. The Chinese translation of the PSQI is validated for sleep quality assessment in China, possessing robust psychometric properties and high test-retest reliability [90].

#### Gaming behavior.

A simple questionnaire developed by our team will be used to collect information on the participants’ weekly engagement in esports gaming. During the intervention period, we will also ask the players to maintain the same frequency of gaming and gaming habits as before.

#### Dietary intake.

During the exercise intervention, participants will be instructed to adhere to their regular diet. To evaluate dietary patterns, a validated food diary assessment will be conducted both before and after the intervention [91]. An online nutrition database (http://www.cfs.gov.hk/) will be used to analyze the dietary records [92].

### Intervention

From previous literature, it has been observed that a HIIT protocol involving 10 × 1-minute bouts at 80–90% of maximum heart rate (HRmax) with 1-minute recovery periods at 40–50% of HRmax is deemed effective [92–94]. Moreover, this intensity and interval have shown significant short-term effects on esports performance in previous studies [27]. This study will follow this protocol for the HIIT exercise group. MICT in this study will consist of continuous exercise at 64–76% of HRmax for 20 minutes, ensuring that the total exercise duration is equivalent to the HIIT protocol to maintain comparable training volumes between groups. A standardized 3-minute warm-up with dynamic stretching and a 3-minute cool-down at 50% HRmax will be added at the beginning and end of each exercise session for both intervention groups. Participants in both intervention groups will be required to complete their respective exercise protocols three times per week throughout the 12-week intervention period. Real-time heart rate monitors (Polar Pacer) will be used to ensure intensity during exercise sessions. In addition, a rating of perceived exertion (RPE) will be employed in each training session utilizing the 6–20 Borg scale [95]. During high-intensity intervals, participants will be instructed to maintain an RPE between 15–18, while during the low-intensity intervals, warm-up, and cool-down phases, an RPE of 11–13 will be targeted [96]. For the MICT group, participants will maintain a moderate-intensity effort corresponding to an RPE of 12–14 throughout the continuous exercise session. Meanwhile, all training sessions will be guided by a professional fitness trainer who will ensure the professionalism and safety of the entire exercise process.

Participants will be allowed to undertake physical exercise either on a treadmill or outdoors, encompassing exercises such as running, jogging, and brisk walking, provided they reach the stipulated heart rate. To account for the potential confounding effect of the exercise environment (indoor vs. outdoor) [97], we will utilize the built-in GPS tracking feature of the Polar Flow system to record the specific setting of each session. This environmental factor will be considered as a covariate in our subsequent statistical analysis. During the initial three sessions, participants will be obliged to exercise in a laboratory under the guidance and supervision of research staff. This ensures not only a precise understanding and execution of the training regimen but also the safety of participants during subsequent unsupervised exercises. Once it is ascertained that participants can meet the training benchmarks without supervision, they are permitted to carry out their exercises at home in a free-living setting for the remainder of the intervention. During these home-based exercises, participants are required to wear a heart rate monitor to track exercise intensity. The monitor records the exercise data throughout the training program, and with prior consent obtained from participants, we will monitor each participant’s exercise data through the *Polar Flow for Coach*, including metrics such as heart rate, frequency, and running speed to confirm participants’ compliance with the exercise plan. Specifically, we define “adherence” using a dual-criterion approach based on both heart rate compliance and session completion. A single training session will be considered “valid” if the participant meets the specific heart rate prescription: for the HIIT group, achieving the target high-intensity zone for at least 80% of the intervals; for the MICT group, maintaining the target heart rate zone for at least 80% of the continuous exercise duration. Consequently, overall adherence to the exercise protocol is defined as the successful completion of at least 80% of the total prescribed valid sessions. This flexible, home-based research design, combined with remote monitoring, aims to further mitigate participant drop-out rates in longer-term interventions and construct an exercise intervention model that can be implemented long-term in a living environment rather than in a laboratory setting, and the methodological approach has also been employed in prior studies [92,98].

### Strategies to improve adherence

Firstly, before enrollment, we repeatedly confirm the participants’ willingness and compliance with the study’s requirements. Secondly, dedicated researchers will be responsible for monitoring the intensity and frequency of the participants’ weekly exercise through the *Polar Flow for Coach* system, and they will repeatedly remind and guide them to complete the 12-week training plan as required. At the same time, the researchers will maintain contact with the participants over the 12 weeks and collect their daily dietary records. Thirdly, participants will receive a supermarket voucher valued at HK$200 upon completion of the intervention.

### Data analysis

An intention-to-treat (ITT) analysis will be conducted as our primary analytical approach to evaluate the overall effectiveness of the intervention. Additionally, a per-protocol analysis will be performed as a sensitivity analysis, which will only include participants who met the predefined adherence criteria. Missing data will be addressed using the Multiple Imputation by Chained Equations (MICE) technique. The normality of the data will be assessed using the Shapiro-Wilk test, and Levene’s Test will be used to assess the homogeneity of variances. Continuous variables will be presented as means with standard deviations (SD) or medians with interquartile ranges (IQR), depending on their data distribution. Categorical variables will be presented as frequencies and percentages. Baseline characteristics among the three groups will be compared using a One-way Analysis of Variance (ANOVA) for normally distributed continuous variables, a Kruskal-Wallis H test for non-normally distributed continuous variables, and Chi-square tests for categorical variables.

To test the main hypothesis, the primary outcome is defined as flicking capacity. Generalized Estimating Equations (GEEs) will be used to determine the group-by-time interaction effect for both the primary and secondary longitudinal outcomes. To rigorously specify the GEE models, the distributional family and link function will be selected based on the specific nature of each outcome variable. An exchangeable working correlation structure will be specified to account for the within-subject correlation of repeated measures across time points. Furthermore, robust standard errors (the sandwich estimator) will be utilized to ensure valid statistical inference even if the working correlation structure is misspecified.

Furthermore, changes in monitored lifestyle variables (i.e., dietary intake, physical activity, and sleep quality) over the intervention period will be evaluated. If any of these variables exhibit significant changes or between-group imbalances, they will be incorporated as covariates in the GEE models to adjust for potential confounding effects, thereby isolating the true effect of the exercise interventions.

The primary outcome will be tested at a two-sided α level of 0.05. For the secondary outcomes, the resulting *p*-values for the group-by-time interaction effects derived from the GEE models will be adjusted using the Benjamini-Hochberg False Discovery Rate (FDR) method. If significant main or interaction effects are detected in the GEE model, simple effect analyses and post-hoc pairwise comparisons will be performed within the GEE framework using estimated marginal means with a Bonferroni adjustment.

Prior to investigating potential associations, we will first test for true inter-individual differences in response to the intervention by comparing the variance of the change between the groups [99]. If true inter-individual variability is confirmed, Pearson’s or Spearman’s rank correlation coefficients will be utilized, as appropriate, to investigate potential associations between esports performance indicators and hand correlates, body composition, and physical competence. To account for multiple testing in these correlation analyses, p-values will be adjusted using the Benjamini-Hochberg FDR method.

All statistical analyses will be conducted using R software (version 4.4.2). A two-sided *p*-value of < 0.05 will be considered statistically significant. To indicate the precision of the estimates, 95% confidence intervals (CIs) will also be calculated and reported.

For the exploratory qualitative component, all semi-structured interviews will be audio-recorded and transcribed verbatim. The transcripts will be analyzed using thematic analysis, facilitated by NVivo 12 software, to identify, analyze, and report recurring themes within the participants’ subjective experiences. Although the sample size is fixed by the intervention design, we will continuously monitor for thematic saturation during this data analysis phase. Rather than formally merging these findings with the quantitative data in a mixed-methods matrix, the qualitative themes will be utilized during the interpretation phase to contextualize the objective performance outcomes. This approach will provide a deeper understanding of the participants’ perspectives and assist in elucidating the potential underlying mechanisms between aerobic exercise and esports performance.

### Data management and monitoring

Data gathered by the measurement software will be individually saved to Excel files immediately following the data-gathering process. Assessments of both physical and cognitive ability will initially be recorded on paper forms devised by the research team, which will subsequently be digitized. The hard copies of these forms will be archived for a decade post-study in distinct filing cabinets. These records will ultimately be compiled into a central database table. All information will be maintained within a safeguarded computer system environment. Access to the folders containing the database will be exclusive to the research personnel. Access will be governed by a secure password system. Prior to analysis, a thorough review of the data will be conducted to ensure accuracy before it is input into analytical software. The ethical guidelines of the study include a comprehensive policy on the handling, labeling, and safeguarding of data, in addition to stipulations on its disclosure and collection.

To maintain privacy, data will be stored under a system of pseudonymization, labeled with unique identification numbers for the trial, and held in restricted-access folders that are accessible solely to the trial team. Any documents with personal identifiers, like names, will be kept separately from the main research files. Password protection will secure all databases at the local level, ensuring controlled access to sensitive information.

### Ethical consideration

This study has been approved by the Survey and Behavioral Research Ethics Committee of The Chinese University of Hong Kong (SBRE‐24‐0942). Participants must sign a written informed consent form before participating in the laboratory experiments and complete an electronic informed consent form before taking the online questionnaire.

Furthermore, we have also enlisted a professional fitness trainer to participate in our experimental process, ensuring the professionalism and safety of our entire exercise program design and fitness assessment. All training sessions will be conducted under the guidance and supervision of these trainers, ensuring the safety of participants in each intervention group throughout the exercise process. Participants are instructed to immediately cease activity if they experience any physical discomfort, thereby preventing potential risks and injuries. In case of an adverse event, we have procedures for immediate assessment, first aid provision, and, if necessary, emergency medical services contact. Incident reporting and follow-up care are standard practices.

## Discussion

This randomized controlled trial seeks to explore the potential benefits of physical exercise on esports performance, compare the differential effects of various exercise modalities, and examine the longitudinal sustainability of these effects. With the rapid expansion of the esports industry and its increasing recognition as a legitimate competitive endeavor, there is a need for more empirical evidence to explore supplementary intervention approaches for esports training and performance enhancement, providing a reference for its scientific development akin to traditional sports. By examining the effects of exercise on the gaming skills, psychomotor, and emotional dimensions of esports performance, this RCT protocol aims to address a critical gap in the current understanding of physical interventions for esports competitors.

### Significance of the study

Although previous studies have demonstrated the potential benefits of physical exercise on esports performance [25–28,30,34,35,56], these investigations are often limited by cross-sectional designs [25,67], acute single-bout exercise interventions [26–28,34], or a reliance on indirect metrics of esports performance [26,30]. While a few longitudinal studies targeting esports performance have recently emerged, they are often constrained by short intervention periods and limited sample sizes. Furthermore, they lack the measurement and analysis of sustained effects post-intervention and have yielded inconsistent findings [31,32]. To better understand the role of physical exercise in esports, a more rigorous evaluation of intervention effects is required. This includes assessing whether exercise can yield long-term performance benefits for players and evaluating the longitudinal sustainability of these effects after the intervention ceases. Furthermore, while much of the current research has predominantly utilized HIIT, further optimizing exercise protocols specifically tailored for esports requires head-to-head comparisons of different exercise modalities. Verifying whether alternative exercise modalities are equally effective will also provide valuable, evidence-based options for decision-makers and coaching staff within esports teams. This protocol is specifically designed to provide a comprehensive research framework that addresses these methodological limitations in the existing literature.

The findings of this study aim to provide empirical insights for esports athletes, coaches, and organizations. The connection between physical fitness and cognitive function has been established in traditional sports, but its application to the context of esports remains under-researched. This study has the potential to demonstrate that physical exercise is not only beneficial for general health but also for the specific demands of esports, such as reaction time, strategic thinking, and endurance during prolonged gaming sessions.

By comparing multiple exercise modalities against a control condition, this study seeks to identify more effective physical training parameters for enhancing esports performance. The inclusion of follow-up assessments extends beyond immediate effects to examine the duration of exercise-induced benefits after training cessation—a critical consideration for establishing optimal training frequency that allows esports athletes to balance physical training with technical practice time. Furthermore, the larger sample size enables us to draw more reliable conclusions about the differential effects of HIIT and MICT in FPS players, thereby offering more specific data for exercise prescription.

Moreover, several negative health-related consequences have been traditionally associated with excessive screen time and prolonged sedentary behavior in video gamers [100,101]. Excessive sitting time and screen exposure pose potential risks to the health of esports athletes [102,103]. Studies have linked prolonged screen time with lower levels of physical activity and poor cardiovascular health [104,105]. Except for active video games or virtual sports, the majority of video games are typically played in a sedentary position [106]. Should the results of the proposed trial demonstrate that physical exercise effectively enhances esports performance, they could offer a practical rationale for integrating systematic, structured physical training into standard esports regimens. Consequently, while optimizing their competitive outcomes, esports players would concurrently reap the well-established health benefits of regular exercise. This integration would effectively mitigate the health risks associated with prolonged sedentary behavior, exploring a potential dual benefit of enhanced competitive performance and safeguarded occupational health.

The comprehensive approach of this study may contribute empirical data to the ongoing discussion regarding physical training in competitive gaming. These findings could serve as a reference for esports academies and professional teams seeking to incorporate appropriate physical exercise regimens alongside technical and tactical training. By integrating physical exercise into training models, this approach aims to address health concerns that could otherwise undermine the longevity of players’ careers, thereby supporting more sustainable practices within competitive gaming.

### Strengths and weaknesses of the study

#### Strengths.

1. The three-arm RCT design (HIIT, MICT, and control) enables not only a robust examination of cause-and-effect relationships between exercise and esports performance, but also direct comparison between different exercise modalities to determine optimal training approaches.
2. The inclusion of follow-up assessments provides unique insights into the sustainability of exercise-induced benefits after intervention cessation, addressing a critical gap in understanding the long-term effects of physical training on esports performance.
3. The larger sample size enhances statistical power, increasing the reliability of findings and enabling more nuanced analyses of intervention effects.
4. This study targets a novel and rapidly growing population, providing timely and relevant data. It utilizes a multi-dimensional approach to measure performance outcomes, including objective measures of gaming skill and subjective measures of mental factors.
5. The comprehensive and structured nature of this protocol offers a clear methodological reference for future interdisciplinary research combining exercise science and esports.

#### Weaknesses.

1. The generalizability of results may be limited due to the specificity of esports genres. In accordance with Chinese gaming regulations, this study excluded minors from participation. This study focuses exclusively on First-Person Shooter games, and findings may not directly translate to other genres such as MOBA or sports games. Future research with larger sample sizes could further analyze the differential effects of interventions across various genres, genders, and age groups among esports players.
2. Attrition rates could be high if participants find the exercise intervention too demanding alongside their regular gaming schedule.
3. The blinding of participants to their intervention group is not feasible due to the nature of exercise interventions, which may introduce some expectation bias in self-reported outcomes.

### Theoretical and practical implications

This RCT is designed to contribute to the esports field by exploring the role of physical exercise as a potential factor in enhancing gaming performance. The three-arm design allows for nuanced comparison between HIIT, MICT, and control conditions, providing enhanced clarity on optimal exercise prescriptions for esports athletes. Should the hypothesis be supported, the findings could encourage a more holistic approach to esports training, emphasizing physical fitness as a core component of their regimen. The results could also encourage further interdisciplinary research that bridges esports with exercise science, psychology, and cognitive neuroscience.

The anticipated outcomes of this protocol may have practical implications across different levels of esports engagement. Within professional contexts, the evidence generated could help refine multidisciplinary training regimens, providing objective data to support the inclusion of physical conditioning alongside technical practice. From an occupational health perspective, empirical support for integrating exercise could help address the physiological risks associated with prolonged sedentary gaming. Furthermore, these findings may not be limited to elite competitors. They could also offer concrete, evidence-based exercise parameters for recreational players seeking to counterbalance the sedentary nature of their habits. Ultimately, by investigating the relationship between specific exercise modalities and esports performance, this protocol aims to provide a scientific foundation for healthier gaming routines, yielding potential benefits for both cognitive performance and long-term well-being.

In conclusion, the design of this protocol aims to address the limitations of existing research regarding the effects of physical exercise on esports performance, providing crucial evidence to further validate the integration of physical activity into esports training. Consequently, this proposed study could contribute to the development of a more comprehensive training approach, offering empirical data on how physical exercise can optimize both performance and well-being in a rapidly professionalizing field. Furthermore, the longitudinal component of this protocol is designed to answer crucial questions about the sustained implementation of exercise, with anticipated implications extending beyond individual player development to potentially inform both future research frameworks combining exercise science and esports and offering practical references for esports training.

## Supporting information

S1 Checklist SPIRIT checklist. SPIRIT 2025 checklist of items to address in a randomized trial protocol. (PDF)

S1 File Ethical protocol. (PDF)

## Abbreviations

DPI: Dots per inch
FDR: False Discovery Rate
FPS: First-Person Shooters
GEE: Generalized Estimating Equation
HIIT: High-Intensity Interval Training
HRmax: Maximum Heart Rate
IPAQ: International Physical Activity Questionnaire
MICE: Multiple Imputation by Chained Equations
MICT: Moderate-Intensity Continuous Training
PACER: Progressive Aerobic Cardiovascular Endurance Run
I-PANAS-SF: International Positive and Negative Affect Schedule Short Form
PAR-Q: Physical Activity Readiness Questionnaire
PSQI: Pittsburgh Sleep Quality Index
RPE: Rating of Perceived Exertion
SMTQ: Sports Mental Toughness Questionnaire
SPIRIT: Standard Protocol Items: Recommendations for Interventional Trials
SRQ: The Self-Regulation Questionnaire
TOPS-2: Test of Performance Strategies-2
VO2max: maximal Oxygen Consumption

## References

[1] Tang D, Sum RK-W, Li M, Ma R, Chung P, Ho RW-K. What is esports? A systematic scoping review and concept analysis of esports. Heliyon. 2023;9(12):e23248. doi: 10.1016/j.heliyon.2023.e23248 38149186 PMC10750068

[2] Formosa J, O’Donnell N, Horton EM, Türkay S, Mandryk RL, Hawks M, et al. Definitions of esports: a systematic review and thematic analysis. Proceedings of the ACM on Human-Computer Interaction. 2022;6(CHI PLAY):227:1-227:45. doi: 10.1145/3549490

[3] Hamari J, Sjöblom M. What is eSports and why do people watch it?. Internet Res. 2017;27(2):211–32.

[4] Block S, Haack F. eSports: a new industry. SHS Web of Conf. 2021;92:04002. doi: 10.1051/shsconf/20219204002

[5] Tang D, Peng B, Ma R, Sum K w a i R. Dispelling stigma and enduring bias: exploring the perception of esports participation among young women. Humanit Soc Sci Commun. 2025;12(1):1–13. doi: 10.1057/s41599-025-04392-z

[6] Hanada Y, Ogasawara E, Inoue Y. Challenges facing the esports industry: a scoping review. Int J Sports Mark Spons. 2025;:1–16. doi: 10.1108/IJSMS-07-2025-0380

[7] Asian Electronic Sports Federation. Asian Electronic Sports Federation. 2019. Available from: https://www.aesf.com/news-media

[8] Esports Charts. 31st Southeast Asian Games ML:BB. 2023. Available from: https://escharts.com/tournaments/mobile-legends/31st-southeast-asian-games

[9] International Esports Federation. Member Nations. 2017. Available from: http://www.ie-sf.org/about/#member-nations

[10] Chiu W, Fan TCM, Nam S-B, Sun P-H. Knowledge Mapping and Sustainable Development of eSports Research: A Bibliometric and Visualized Analysis. Sustainability. 2021;13(18):10354. doi: 10.3390/su131810354

[11] Pluss MA, Bennett KJM, Novak AR, Panchuk D, Coutts AJ, Fransen J. Esports: The Chess of the 21st Century. Front Psychol. 2019;10:156. doi: 10.3389/fpsyg.2019.00156 30761055 PMC6363684

[12] Sanz-Matesanz M, Gea-García GM, Martínez-Aranda LM. Physical and psychological factors related to player’s health and performance in esports: A scoping review. Comput Hum Behav. 2023;107698.

[13] Pedraza-Ramirez I, Sharpe BT, Behnke M, Toth AJ, Poulus DR. The psychology of esports: Trends, challenges, and future directions. Psychol Sport Exerc. 2025;81:102967. doi: 10.1016/j.psychsport.2025.102967 40774402

[14] Colcombe S, Kramer AF. Fitness effects on the cognitive function of older adults: a meta-analytic study. Psychol Sci. 2003;14(2):125–30. doi: 10.1111/1467-9280.t01-1-01430 12661673

[15] Nordin AD, Rymer WZ, Biewener AA, Schwartz AB, Chen D, Horak FB. Biomechanics and neural control of movement, 20 years later: what have we learned and what has changed?. J NeuroEngineering Rehabil. 2017;14(1):91. doi: 10.1186/s12984-017-0298-y PMC5594571 28893279

[16] Chang Y-K, Ren F-F, Li R-H, Ai J-Y, Kao S-C, Etnier JL. Effects of acute exercise on cognitive function: A meta-review of 30 systematic reviews with meta-analyses. Psychol Bull. 2025;151(2):240–59. doi: 10.1037/bul0000460 39883421

[17] Bediou B, Adams DM, Mayer RE, Tipton E, Green CS, Bavelier D. Meta-analysis of action video game impact on perceptual, attentional, and cognitive skills. Psychol Bull. 2018;144(1):77–110. doi: 10.1037/bul0000130 29172564

[18] Granic I, Lobel A, Engels RCME. The benefits of playing video games. Am Psychol. 2014;69(1):66–78. doi: 10.1037/a0034857 24295515

[19] Egawa J, Hochi Y, Iwaasa T, Togashi E, Inaba K, Mizuno M. The Effects of University Students’ Physical Activity Experience on Communication Skills and Anxiety. Advances in Intelligent Systems and Computing. Springer International Publishing. 2019. 358–66. 10.1007/978-3-030-20145-6_36

[20] Özdenk S, İmamoğlu M. The Effects of Pilates, Step and Zumba Exercises on Self-esteem, Happiness and Communication Skill Levels. Asian Journal of Education and Training. 2019;5(2):369–73. doi: 10.20448/journal.522.2019.52.369.373

[21] Smith PJ, Blumenthal JA, Hoffman BM, Cooper H, Strauman TA, Welsh-Bohmer K, et al. Aerobic exercise and neurocognitive performance: a meta-analytic review of randomized controlled trials. Psychosom Med. 2010;72(3):239–52. doi: 10.1097/PSY.0b013e3181d14633 20223924 PMC2897704

[22] Nagorsky E, Wiemeyer J. The structure of performance and training in esports. PLoS One. 2020;15(8):e0237584. doi: 10.1371/journal.pone.0237584 32841263 PMC7447068

[23] Sörman DE, Dahl KE, Lindmark D, Hansson P, Vega-Mendoza M, Körning-Ljungberg J. Relationships between Dota 2 expertise and decision-making ability. PLoS One. 2022;17(3):e0264350. doi: 10.1371/journal.pone.0264350 35231043 PMC8887737

[24] Mancı E, Güdücü Ç, Günay E, Güvendi G, Campbell M, Bediz CŞ. The relationship between esports game genres and cognitive performance: A comparison between first-person shooter vs. multiplayer online battle arena games in younger adults. Ent Comput. 2024;50:100640. doi: 10.1016/j.entcom.2024.100640

[25] Tang D, Ma R, Chung P, Ho W keung, Sum K wai R. Synergistic fields: Unveiling the potential win-win relationship between esports performance and traditional sports participation. PLOS ONE. 2024;19(8):e0305880. doi: 10.1371/journal.pone.0305880 PMC11318873 39133747

[26] DiFrancisco-Donoghue J, Jenny SE, Douris PC, Ahmad S, Yuen K, Hassan T, et al. Breaking up prolonged sitting with a 6 min walk improves executive function in women and men esports players: a randomised trial. BMJ Open Sport Exerc Med. 2021;7(3):e001118. doi: 10.1136/bmjsem-2021-001118

[27] deE Las Heras B, Li O, Rodrigues L, Nepveu J-F, Roig M. Exercise Improves Video Game Performance: A Win-Win Situation. Med Sci Sports Exerc. 2020;52(7):1595–602. doi: 10.1249/MSS.0000000000002277 31977638

[28] Rightmire ZB, Agostinelli PJ, Murrah WM, Roper JA, Roberts MD, Sefton JM. Acute high-intensity interval training improves esport performance in Super Smash Brothers Ultimate competitors. J Electron Gaming Esports. 2024;2(1). doi: 10.1123/jege.2023-0031

[29] McNulty C, Jenny SE, Leis O, Poulus D, Sondergeld P, Nicholson M. Physical exercise and performance in esports players: an initial systematic review. J Electron Gaming Esports. 2023;1(1). doi: 10.1123/jege.2022-0014

[30] Sanz-Matesanz M, Martínez-Aranda LM, Gea-García GM. Effects of a physical training program on cognitive and physical performance and health-related variables in professional esports players: A pilot study. Applied Sciences. 2024;14(7):7. doi: 10.3390/app14072845

[31] Wachholz F, Gamper N, Schnitzer M. An 8-week physical exercise intervention for e’athletes improves physical performance rather than short-term esports performance parameters - a randomized controlled trial. Front Sports Act Living. 2025;6:1504205. doi: 10.3389/fspor.2024.1504205 39906318 PMC11790659

[32] Tang D, Zhang X, Ho RW-K, Choi SM, Ye Z, Campbell MJ, et al. Effect of Physical Exercise on Esports Performance in First-Person Shooter Gamers: A Six-Week Randomized Controlled Trial. Sports Med Open. 2025;11(1):162. doi: 10.1186/s40798-025-00970-2 41444460 PMC12738490

[33] Tang D. Exploration of esports on its participation, health and performance: a mixed-methods study. 2025. 10.1136/bjsports-2025-109820 40147867

[34] Dos Santos J, Figuireido T, Benoît CE, Chaplais E. Physical and/or cognitive warm-up effects on first-person shooter video-games performances. Entertainment Computing. 2024;49:100618. doi: 10.1016/j.entcom.2023.100618

[35] Zhang W, Wang X, Li X, Yan H, Song Y, Li X, et al. Effects of acute moderate-intensity aerobic exercise on cognitive function in E-athletes: A randomized controlled trial. Med. 2023;102(40):e35108. doi: 10.1097/MD.0000000000035108 PMC10553036 37800783

[36] Grace F, Herbert P, Elliott AD, Richards J, Beaumont A, Sculthorpe NF. High intensity interval training (HIIT) improves resting blood pressure, metabolic (MET) capacity and heart rate reserve without compromising cardiac function in sedentary aging men. Exp Gerontol. 2018;109:75–81. doi: 10.1016/j.exger.2017.05.010 28511954

[37] Weston M, Taylor KL, Batterham AM, Hopkins WG. Effects of low-volume high-intensity interval training (HIT) on fitness in adults: a meta-analysis of controlled and non-controlled trials. Sports Med. 2014;44(7):1005–17. doi: 10.1007/s40279-014-0180-z 24743927 PMC4072920

[38] Ludyga S, Gerber M, Brand S, Holsboer-Trachsler E, Pühse U. Acute effects of moderate aerobic exercise on specific aspects of executive function in different age and fitness groups: A meta-analysis. Psychophysiology. 2016;53(11):1611–26. doi: 10.1111/psyp.12736 27556572

[39] Erickson KI, Voss MW, Prakash RS, Basak C, Szabo A, Chaddock L, et al. Exercise training increases size of hippocampus and improves memory. Proceedings of the National Academy of Sciences. 2011;108(7):3017–22. doi: 10.1073/pnas.1015950108 PMC3041121 21282661

[40] McMorris T, Hale BJ. Differential effects of differing intensities of acute exercise on speed and accuracy of cognition: a meta-analytical investigation. Brain Cogn. 2012;80(3):338–51. doi: 10.1016/j.bandc.2012.09.001 23064033

[41] Kamijo K, Hayashi Y, Sakai T, Yahiro T, Tanaka K, Nishihira Y. Acute Effects of Aerobic Exercise on Cognitive Function in Older Adults. J Gerontol Ser B. 2009;64B(3):356–63. doi: 10.1093/geronb/gbp030 19363089

[42] Calverley TA, Ogoh S, Marley CJ, Steggall M, Marchi N, Brassard P, et al. HIITing the brain with exercise: mechanisms, consequences and practical recommendations. J Physiol. 2020;598(13):2513–30. doi: 10.1113/JP275021 32347544

[43] Carter JB, Banister EW, Blaber AP. Effect of endurance exercise on autonomic control of heart rate. Sports Med. 2003;33(1):33–46. doi: 10.2165/00007256-200333010-00003 12477376

[44] Albinet CT, Boucard G, Bouquet CA, Audiffren M. Increased heart rate variability and executive performance after aerobic training in the elderly. Eur J Appl Physiol. 2010;109(4):617–24. doi: 10.1007/s00421-010-1393-y 20186426

[45] de Lima NS, De Sousa RAL, Amorim FT, Gripp F, Diniz E Magalhães CO, Henrique Pinto S, et al. Moderate-intensity continuous training and high-intensity interval training improve cognition, and BDNF levels of middle-aged overweight men. Metab Brain Dis. 2022;37(2):463–71. doi: 10.1007/s11011-021-00859-5 34762211

[46] Jung B-K, Kim K. Effects of 12 Weeks of Moderate-intensity Continuous Exercise and High-intensity Interval Exercise on Cognitive Function in Elderly Subjects. Asian J Kinesiol. 2024;26(2):48–58. doi: 10.15758/ajk.2024.26.2.48

[47] Amorim Oliveira GT, Elsangedy HM, Pereira DC, de Melo Silva R, Campos Faro HK, Bortolotti H, et al. Effects of 12 weeks of high-intensity interval, moderate-intensity continuous and self-selected intensity exercise training protocols on cognitive inhibitory control in overweight/obese adults: A randomized trial. Eur J Sport Sci. 2022;22(11):1724–33. doi: 10.1080/17461391.2021.1969433 34429030

[48] Mekari S, Earle M, Martins R, Drisdelle S, Killen M, Bouffard-Levasseur V, et al. Effect of High Intensity Interval Training Compared to Continuous Training on Cognitive Performance in Young Healthy Adults: A Pilot Study. Brain Sci. 2020;10(2):81. doi: 10.3390/brainsci10020081 32033006 PMC7071608

[49] Coetsee C, Terblanche E. The effect of three different exercise training modalities on cognitive and physical function in a healthy older population. Eur Rev Aging Phys Act. 2017;14(1). doi: 10.1186/s11556-017-0183-5 PMC5553712 28811842

[50] Chang YK, Labban JD, Gapin JI, Etnier JL. The effects of acute exercise on cognitive performance: a meta-analysis. Brain Res. 2012;1453:87–101. doi: 10.1016/j.brainres.2012.02.068 22480735

[51] Deleuze J, Christiaens M, Nuyens F, Billieux J. Shoot at first sight! First person shooter players display reduced reaction time and compromised inhibitory control in comparison to other video game players. Comput Hum Behav. 2017;72:570–6. doi: 10.1016/j.chb.2017.02.027

[52] Iwatsuki T, Hagiwara G, Dugan ME. Effectively optimizing esports performance through movement science principles. International Journal of Sports Science & Coaching. 2021;17(1):202–7. doi: 10.1177/17479541211016927

[53] Matuszewski P, Dobrowolski P, Zawadzki B. The Association Between Personality Traits and eSports Performance. Front Psychol. 2020;11:1490. doi: 10.3389/fpsyg.2020.01490 32754085 PMC7366824

[54] Pluss MA, Novak AR, Bennett KJM, Panchuk D, Coutts AJ, Fransen J. The reliability and validity of mobalytics proving ground as a perceptual-motor skill assessment for esports. International Journal of Sports Science & Coaching. 2022;18(2):470–9. doi: 10.1177/17479541221086793

[55] Toth AJ, Hojaji F, Campbell MJ. Exploring the mechanisms of target acquisition performance in esports: The role of component kinematic phases on a first person shooter motor skill. Comput Hum Behav. 2023;139:107554. doi: 10.1016/j.chb.2022.107554

[56] Dykstra R, Koutakis P, Hanson N. Relationship Between Physical Fitness Variables and Reaction Time in eSports Gamers. International Journal of eSports Research. 2021;1(1):1–14. doi: 10.4018/ijer.288540

[57] Timokhov V, Sergey S. Cognitive styles are poor predictors of esports player’s performance in League of Legends. 2023.

[58] Toth AJ, Kowal M, Campbell MJ. The color-word Stroop task does not differentiate cognitive inhibition ability among esports gamers of varying expertise. Frontiers in Psychology. 2019. doi: 10.3389/fpsyg.2019.02852 PMC6932966 31920879

[59] Poulus D, Coulter TJ, Trotter MG, Polman R. Stress and Coping in Esports and the Influence of Mental Toughness. Frontiers in Psychology. 2020;11. 10.3389/fpsyg.2020.00628 PMC7191198 32390900

[60] Smith MJ, Birch PDJ, Bright D. Identifying Stressors and Coping Strategies of Elite Esports Competitors. International Journal of Gaming and Computer-Mediated Simulations. 2019;11(2):22–39. doi: 10.4018/ijgcms.2019040102

[61] Office of the Government Chief Information Officer HKSAR. Report on Promotion of E-sports Development in Hong Kong. 2018. Available from: https://www.legco.gov.hk/yr17-18/chinese/panels/itb/papers/itb20171211cb4-608-1-c.pdf

[62] Wong MYC, Chung P-K, Ou K, Leung K-M. Perception of Hong Kong Teenagers and Young Adults on Esports Participation: A Qualitative Study Using Theory of Planned Behavior. Front Psychol. 2021;12:650000. doi: 10.3389/fpsyg.2021.650000 34305718 PMC8299725

[63] General Administration of Press and Publication of The People’s Republic of China. Implementation of online Game Fatigue System. 2007. Available from: http://www.gov.cn/zwgk/2007-04/13/content_581444.htm

[64] Green CS, Bavelier D. Action video game modifies visual selective attention. Nature. 2003;423(6939):534–7. doi: 10.1038/nature01647 12774121

[65] Warburton DER, Jamnik VK, Bredin SSD, Gledhill N. The Physical Activity Readiness Questionnaire for Everyone (PAR-Q) and Electronic Physical Activity Readiness Medical Examination (ePARmed-X). Health Fit J Can. 2011;4(2):3–17. doi: 10.14288/hfjc.v4i2.103

[66] Ross R, Hudson R, Stotz PJ, Lam M. Effects of exercise amount and intensity on abdominal obesity and glucose tolerance in obese adults. Ann Intern Med. 2015;162(5):325–34. doi: 10.7326/M14-1189 25732273

[67] Tang D, Gou P, Liu S, Zhang X, Li D, Liu J. Effects of unsupervised campus HIIT on fitness and sleep in sedentary male students: a randomized controlled trial. BMC Public Health. 2026. doi: 10.1186/s12889-026-27310-7 PMC13195952 41957758

[68] Lamers James RG, O’Connor AR. Impact of focus of attention on aiming performance in the first-person shooter videogame Aim Lab. PLoS One. 2023;18(7):e0288937. doi: 10.1371/journal.pone.0288937 37490480 PMC10368276

[69] Roldan CJ, Prasetyo YT. Evaluating The Effects of Aim Lab Training on Filipino Valorant Players’ Shooting Accuracy. In: 2021 IEEE 8th International Conference on Industrial Engineering and Applications (ICIEA), 2021. 465–70. 10.1109/iciea52957.2021.9436822

[70] Brown JM. Self-Regulation and the Addictive Behaviors. Treating Addictive Behaviors. Springer US. 1998. p. 61–73. 10.1007/978-1-4899-1934-2_5

[71] Carey KB, Neal DJ, Collins SE. A psychometric analysis of the self-regulation questionnaire. Addict Behav. 2004;29(2):253–60. doi: 10.1016/j.addbeh.2003.08.001 14732414

[72] Hardy L, Roberts R, Thomas PR, Murphy SM. Test of Performance Strategies (TOPS): Instrument refinement using confirmatory factor analysis. Psychology of Sport and Exercise. 2010;11(1):27–35. doi: 10.1016/j.psychsport.2009.04.007

[73] Sheard M, Golby J, Van Wersch P. Progress toward construct validation of the sports mental toughness questionnaire (SMTQ). EUR J PSYCHOL ASSESS. 2009;25(3):186–93. doi: 10.1027/1015-5759.25.3.186

[74] Trotter MG, Coulter TJ, Davis PA, Poulus DR, Polman R. Social support, self-regulation, and psychological skill use in e-athletes. Frontiers in Psychology. 2021;12. 10.3389/fpsyg.2021.722030 PMC8632024 34858261

[75] Gao J, Xiang J, Hou Z, Liu H. Relationship between training status and stress response in Chinese college student-athletes: chain mediation between sport performance strategies and coping styles. Front Psychol. 2025;16:1597539. doi: 10.3389/fpsyg.2025.1597539 40703744 PMC12285528

[76] Chen Y-H, Lin Y-J. Validation of the Short Self-Regulation Questionnaire for Taiwanese College Students (TSSRQ). Front Psychol. 2018;9:259. doi: 10.3389/fpsyg.2018.00259 29551987 PMC5840206

[77] Gu S, Xue L. Relationships among sports group cohesion, psychological collectivism, mental toughness and athlete engagement in Chinese team sports athletes. Int J Environ Res Public Health. 2022;19(9):4987. doi: 10.3390/ijerph19094987 35564390 PMC9103217

[78] Mayorga-Vega D, Aguilar-Soto P, Viciana J. Criterion-Related Validity of the 20-M Shuttle Run Test for Estimating Cardiorespiratory Fitness: A Meta-Analysis. J Sports Sci Med. 2015;14(3):536–47. 26336340 PMC4541117

[79] Léger LA, Mercier D, Gadoury C, Lambert J. The multistage 20 metre shuttle run test for aerobic fitness. J Sports Sci. 1988;6(2):93–101. doi: 10.1080/02640418808729800 3184250

[80] Roberts HC, Denison HJ, Martin HJ, Patel HP, Syddall H, Cooper C, et al. A review of the measurement of grip strength in clinical and epidemiological studies: towards a standardised approach. Age Ageing. 2011;40(4):423–9. doi: 10.1093/ageing/afr051 21624928

[81] Strand SL, Hjelm J, Shoepe TC, Fajardo MA. Norms for an isometric muscle endurance test. J Hum Kinet. 2014;40:93–102. doi: 10.2478/hukin-2014-0011 25031677 PMC4096102

[82] Springer BA, Marin R, Cyhan T, Roberts H, Gill NW. Normative values for the unipedal stance test with eyes open and closed. J Geriatr Phys Ther. 2007;30(1):8–15. doi: 10.1519/00139143-200704000-00003 19839175

[83] Pauole K, Madole K, Garhammer J, Lacourse M, Rozenek R. Reliability and Validity of the T-Test as a Measure of Agility, Leg Power, and Leg Speed in College-Aged Men and Women. Journal of Strength and Conditioning Research. 2000;14(4):443–50. doi: 10.1519/00124278-200011000-00012

[84] Rikli RE, Jones CJ. Development and validation of a functional fitness test for community-residing older adults. J Aging Phys Act. 1999;7(2):129–61. doi: 10.1123/japa.7.2.129

[85] Thompson ER. Development and Validation of an Internationally Reliable Short-Form of the Positive and Negative Affect Schedule (PANAS). Journal of Cross-Cultural Psychology. 2007;38(2):227–42. doi: 10.1177/0022022106297301

[86] Liu J-D, You R-H, Liu H, Chung P-K. Chinese version of the international positive and negative affect schedule short form: factor structure and measurement invariance. Health Qual Life Outcomes. 2020;18(1):285. doi: 10.1186/s12955-020-01526-6 32838809 PMC7447563

[87] Craig CL, Marshall AL, Sjöström M, Bauman AE, Booth ML, Ainsworth BE, et al. International physical activity questionnaire: 12-country reliability and validity. Med Sci Sports Exerc. 2003;35(8):1381–95. doi: 10.1249/01.MSS.0000078924.61453.FB 12900694

[88] Macfarlane D, Chan A, Cerin E. Examining the validity and reliability of the Chinese version of the International Physical Activity Questionnaire, long form (IPAQ-LC). Public Health Nutr. 2011;14(3):443–50. doi: 10.1017/S1368980010002806 20939939

[89] Buysse DJ, Reynolds CF 3rd, Monk TH, Berman SR, Kupfer DJ. The Pittsburgh Sleep Quality Index: a new instrument for psychiatric practice and research. Psychiatry Res. 1989;28(2):193–213. doi: 10.1016/0165-1781(89)90047-4 2748771

[90] Tsai P-S, Wang S-Y, Wang M-Y, Su C-T, Yang T-T, Huang C-J, et al. Psychometric Evaluation of the Chinese Version of the Pittsburgh Sleep Quality Index (CPSQI) in Primary Insomnia and Control Subjects. Qual Life Res. 2005;14(8):1943–52. doi: 10.1007/s11136-005-4346-x 16155782

[91] Chinnock A. Validation of an estimated food record. Public Health Nutr. 2006;9(7):934–41. doi: 10.1017/phn2005922 17010260

[92] Poon ET-C, Little JP, Sit CH-P, Wong SH-S. The effect of low-volume high-intensity interval training on cardiometabolic health and psychological responses in overweight/obese middle-aged men. J Sports Sci. 2020;38(17):1997–2004. doi: 10.1080/02640414.2020.1766178 32497454

[93] Poon ET-C, Siu PM-F, Wongpipit W, Gibala M, Wong SH-S. Alternating high-intensity interval training and continuous training is efficacious in improving cardiometabolic health in obese middle-aged men. J Exerc Sci Fit. 2022;20(1):40–7. doi: 10.1016/j.jesf.2021.11.003 34987589 PMC8689221

[94] Wen D, Utesch T, Wu J, Robertson S, Liu J, Hu G, et al. Effects of different protocols of high intensity interval training for VO2max improvements in adults: A meta-analysis of randomised controlled trials. J Sci Med Sport. 2019;22(8):941–7. doi: 10.1016/j.jsams.2019.01.013 30733142

[95] Borg G. Borg’s perceived exertion and pain scales. Human Kinetics. 1998. Available from: https://psycnet.apa.org/record/1998-07179-000

[96] Taylor JL, Holland DJ, Keating SE, Leveritt MD, Gomersall SR, Rowlands AV, et al. Short-term and Long-term Feasibility, Safety, and Efficacy of High-Intensity Interval Training in Cardiac Rehabilitation: The FITR Heart Study Randomized Clinical Trial. JAMA Cardiol. 2020;5(12):1382–9. doi: 10.1001/jamacardio.2020.3511 32876655 PMC7489382

[97] Walters G, Dring KJ, Williams RA, Needham R, Cooper SB. Outdoor physical activity is more beneficial than indoor physical activity for cognition in young people. Physiol Behav. 2025;295:114888. doi: 10.1016/j.physbeh.2025.114888 40120965

[98] Vella CA, Taylor K, Drummer D. High-intensity interval and moderate-intensity continuous training elicit similar enjoyment and adherence levels in overweight and obese adults. Eur J Sport Sci. 2017;17(9):1203–11. doi: 10.1080/17461391.2017.1359679 28792851 PMC6104631

[99] Atkinson G, Batterham AM. True and false interindividual differences in the physiological response to an intervention. Exp Physiol. 2015;100(6):577–88. doi: 10.1113/EP085070 25823596

[100] Duncan MJ, Vandelanotte C, Caperchione C, Hanley C, Mummery WK. Temporal trends in and relationships between screen time, physical activity, overweight and obesity. BMC Public Health. 2012;12:1060. doi: 10.1186/1471-2458-12-1060 23216917 PMC3541208

[101] Owen N, Healy GN, Matthews CE, Dunstan DW. Too much sitting: the population health science of sedentary behavior. Exerc Sport Sci Rev. 2010;38(3):105–13. doi: 10.1097/JES.0b013e3181e373a2 20577058 PMC3404815

[102] Mi Y, Zhao S, Ju F. An integrated health management model to improve the health of professional e-sports athletes: a literature review. PeerJ. 2025;13:e19323. doi: 10.7717/peerj.19323 40276298 PMC12020735

[103] Soffner M, Schmidt A, Tomschi F, Hilberg T. Do esports players experience pain? Pain prevalence of esports players: a systematic review and meta-analysis. Sports Med - Open. 2026;12(1):1. doi: 10.1186/s40798-025-00971-1 41483384 PMC12764727

[104] Archer E, Blair SN. Physical activity and the prevention of cardiovascular disease: from evolution to epidemiology. Prog Cardiovasc Dis. 2011;53(6):387–96. doi: 10.1016/j.pcad.2011.02.006 21545924

[105] Weaver JB, Mays D, Sargent Weaver S, Kannenberg W, Hopkins GL, Eroğlu D, et al. Health-risk correlates of video-game playing among adults. Am J Prev Med. 2009;37(4):299–305. doi: 10.1016/j.amepre.2009.06.014 19765501

[106] Graves L, Stratton G, Ridgers ND, Cable NT. Comparison of energy expenditure in adolescents when playing new generation and sedentary computer games: cross sectional study. BMJ. 2007;335(7633):1282–4. doi: 10.1136/bmj.39415.632951.80 18156227 PMC2151174

