# Supplementary material for: Can different exercise modalities improve esports performance? Study protocol for a 12-week three-arm randomized controlled trial
Source: PLoS One. 2026 Aug 25;21(8):e0357119. doi: 10.1371/journal.pone.0357119 (PMC13505923; doi:10.1371/journal.pone.0357119)
Supplement: Supplementary file 2 [file pone.0357119.s002.pdf]

Application Reference No.	SBRE-24-0942
Application Status	Application Approved

PI and Project Information

Principal Investigator (PI)	YE Zheng
Preferred Name of Principal Investigator (PI)	Mr. YE Zheng
PI Title / Position	PhD Student
Survey and Behavioral Ethics Training Cert. Number	S84706675 (Valid through 2028-02-23)
Publication Ethics Training Cert. Number	P14409202 (Valid through 2028-02-23)
Supervisor(O365 Login ID)	kwsum@cuhk.edu.hk (Professor SUM Kim Wai Raymond)
Programme Code	EDU-F-PHD
Programme Name	PhD Education
Department/Unit	Faculty of Education

Project Title

Investigation of the Effects of Different Exercise Modalities on Esports Performance: A 12-Week Three-Arm Randomized Controlled Trial

---

Estimated Start Date of the Study	2026-01-01
Reason for Late Submission	
Estimated End Date of the Study	2028-12-31
Name of Grant/Funding (if applicable)	
Reference No. of Grant/Funding (if applicable)	
Amount Applying / Approved	
Status of Grant/Funding	○ Approved<br>○ Pending<br>◉ Not Applicable
A copy of your grant proposal (if applicable)	

Summary of Research Proposal

Summarize, in layperson's language, the below details of the study with no more than 200 words per each:

(1) Objective(s)

This study aims to investigate the long-term effects of high-intensity interval training (HIIT) and moderate-intensity continuous training (MICT) on esports performance through a 12-week three-arm randomized controlled trial, and to examine the sustainability of these effects during a follow-up period after intervention cessation. Additionally, we seek to compare the differential impacts of these two exercise modalities on various dimensions of esports performance, thereby providing evidence-based guidance for esports training regimens and enhancing our understanding of the underlying mechanisms by which different exercise approaches influence cognitive and motor capabilities essential for competitive gaming.

---

(2) Significance

This research investigates the differential effects of High-Intensity Interval Training (HIIT) and Moderate-Intensity Continuous Training (MICT) on esports performance. While prior research suggests exercise benefits cognitive function crucial for gaming, no study has directly compared these modalities for competitive gamers. This 12-week intervention compares HIIT, MICT, and a control group among FPS players, addressing this gap. This study extends our previous research on exercise and esports performance, which found no significant improvement in psychological attributes like mental toughness and self-regulation, potentially due to insufficient intensity and duration. This study addresses these limitations with a larger sample size, longer intervention, and diverse exercise protocols. By comparing the three groups, this randomized controlled trial will provide the first direct evidence of how different training modalities impact specific gaming performance aspects. The findings will inform evidence-based training regimens for esports organizations and contribute to understanding the exercise-cognition-performance relationship in competitive gaming.

---

(3) Research Methodology(ies)

We will recruit healthy adult first-person shooter gamers for this study through mass mail websites of the university and social media platforms such as WeChat groups, Instagram, etc. This study will be designed as a three-arm randomized controlled trial. 195 participants (dropout rate considered) will be randomly allocated into three groups (1:1:1 ratio): HIIT group, MICT group, and control group. A computer-based randomization tool (https://www.randomizer.org) will be utilized to facilitate random allocation. The entire allocation process will be conducted by a staff member who will not be participating in the study. The HIIT group will receive a 12-week HIIT exercise program, while the MICT group will participate in a 12-week MICT program. The control group will not receive any exercise intervention. All groups will report the duration of their weekly self-initiated exercise and

engagement in esports. Selected participants will undergo a baseline test (week 0), followed by a post-test after the 12-week intervention period (week 12), and a follow-up assessment 4 weeks after the intervention completion (week 16) to examine the sustainability of effects after training cessation.

**Participant Information Sheet or Consent Form**	Consent Form (15).docx (0.02MB)
**Questionnaires and measures**	Survey (2).docx (0.01MB)

Type of Study

Type of Study

- [ ] This research will be conducted in established or commonly accepted educational settings, involving normal educational practices that are not likely to adversely impact students' opportunity to learn or assessment of educators. All participants are **adults**.
- [ ] This research will involve the use of educational tests (cognitive, diagnostic, aptitude, achievement), survey procedures, interview procedures or observation of public behaviour of **adult** participants. Recorded information cannot readily identify the participant (directly or indirectly/linked), OR any disclosure of responses outside of the research would NOT reasonably place participant at risk (criminal, civil liability, financial, employability, educational advancement, reputation).
- [ ] This research will involve benign behavioural interventions through verbal, written responses, (including data entry or audiovisual recording) from **adult** participants who agrees. Recorded information cannot readily identify the participant (directly or indirectly/linked), OR any disclosure of responses outside of the research would NOT reasonably place participant at risk (criminal, civil liability, financial, employability, educational advancement, reputation).
- [ ] This research is a secondary research project with identifiable information collected for some other initial activity, if information is publicly available, OR information is recorded in such a way that participant cannot readily be identified (directly or indirectly/linked); investigator does not contact participants and will not re-identify the participants.
- [ ] This is research on individual or group characteristics or behaviour (including, but not limited to, research on perception, cognition, motivation, identity, language, communication, cultural beliefs or practices, and social behaviour) or research employing survey, interview, oral history, focus group, program evaluation, human factors evaluation, or quality assurance methodologies. Recorded information cannot readily identify the participant (directly or indirectly/linked), OR any disclosure of responses outside of the research would NOT reasonably place participant at risk (criminal, civil liability, financial, employability, educational advancement, reputation). All participants are **adults**.
- [x] Not applicable.

Participants

**Is there any participant involved?**	(•) Yes ( ) No	
**Number of Healthy Adults:**	195	
**Number of Children (under 18 years old):**	0	
**Number of Pregnant Women:**	0	
**Number of Outpatients:**	0	
**Number of Inpatients:**	0	
**Number of Prisoners:**	0	
**Number of Cognitively Impaired Persons, please specify**	0	0
**Others, please specify**	0	0
**Total Number of Participants:**	195	

Inclusion / Exclusion Criteria

Breakdown of the planned number of participants

**Number of Male Adult:**	97
**Number of Female Adult:**	98
**Number of Children:**	0
**Lower age limit of the participants (0 to indicate no limit):**	18

Upper age limit of the participants (999 to indicate no limit): 29

Are there any participant recruitment restrictions based on race/ethnicity of the participant?

- ○ Yes
- ◉ No

What are the inclusion criteria in participant recruitment?

a. 18-29 years old; b. regular FPS players engaging in shooters within the past six months; c. healthy individuals who are capable of physical tests or HIIT; d. no regular exercise habits; e. can understand English or Chinese.

What are the exclusion criteria in participant recruitment?

a. self-reported history of neurological, psychiatric, or medical diseases; b. current intake of medications and/or recreational drugs that could affect the central nervous system and/or the ability to learn; c. not a regular user of Aim Lab.

Are the participants in a dependent relationship with the researchers (e.g., researchers' children or students)

- ○ Yes, please provide details:
- ◉ No

Participant Recruitment

Describe how the participants will be recruited with no more than 100 words

We will recruit healthy adult first-person shooter gamers for this study through mass mail websites of the university and social media platforms such as WeChat groups, Instagram, etc. Additionally, the QR code for the initial online survey will be displayed at offline esports activities for anyone who is interested in participating.

Advertisement used to recruit participants for your research (if applicable)

Tasks / Activities Performed by the Participants

Task 1 of 2

Description of task / activity to be included

Duration: Each session consists of 10 x 1-minute high-intensity bouts interspersed with 10 x 1-minute recovery periods. Adding the 3-minute warm-up and 3-minute cool-down, each session totals approximately 26 minutes. This protocol is followed for 12 weeks. Intensity: High-intensity bouts are performed at 80-90% of HRmax (RPE 15-18). Recovery periods are at 40-50% of HRmax (RPE 11-13). Warm-up and cool-down are at 50% HRmax (RPE 11-13). Modality: Running, jogging, or brisk walking, either on a treadmill or outdoors. Supervision: The first three sessions are supervised in a laboratory setting. Subsequent sessions are unsupervised and performed at home with heart rate monitoring and remote data tracking via Polar Flow for Coach.

Target participants of this task / activity: 65

Duration of participants to be involved in this task / activity: 26 minutes

Indicate the number of times / research visits the participant needs to make to complete this task / activity: 12visits

Questionnaires and measures that will be used in the study: Measurement (3).docx (0.24MB)

Risk Level

- ◉ Minimal risk
- ○ More than minimal risk (describe the risk)

Risk Description

Our test does not include any invasive procedures or tests. To ensure the safety of participants throughout the physical fitness testing and exercise intervention, participants will undergo a screening process to determine if they are physically and mentally capable of participating in the testing and exercise. First-time participants may experience some fatigue after the exercise. The entire process will be accompanied by qualified first aid personnel and sports professionals. At the same time, participants in the intervention group will receive safety knowledge training to ensure that they can complete the exercise safely at home. Participants can stop immediately and inform the researchers if they experience any physical or mental discomfort during exercise.

The PI is responsible for ensuring that all research participants give informed consent before enrolling into the research.

Do you request a waiver of informed consent?

- ○ Yes
- ◉ No

Please upload Participant Information Sheet or Consent Form.

Consent Form (15).docx (0.02MB)

Please upload Assent Form (if applicable).

Task 2 of 2

Description of task / activity to be included

Duration: Each session consists of 20 minutes of continuous exercise. Including the 3-minute warm-up and 3-minute cool-down, each session totals 26 minutes. This protocol is followed for 12 weeks. Intensity: Exercise is performed at 64-76% of HRmax (RPE 12-14). Warm-up and cool-down are at 50% HRmax (RPE 11-13). Modality: Running, jogging, or brisk walking, either on a treadmill or outdoors. Supervision: The first three sessions are supervised in a laboratory setting. Subsequent sessions are unsupervised and performed at home with heart rate monitoring and remote data tracking via Polar Flow for Coach.

Target participants of this task / activity 65

Duration of participants to be involved in this task / activity 26 minutes

Indicate the number of times / research visits the participant needs to make to complete this task / activity 12visits

Questionnaires and measures that will be used in the study Measurement (3).docx (0.24MB)

Risk Level

- ◉ Minimal risk
- ○ More than minimal risk (describe the risk)

Risk Description

Our test does not include any invasive procedures or tests. To ensure the safety of participants throughout the physical fitness testing and exercise intervention, participants will undergo a screening process to determine if they are physically and mentally capable of participating in the testing and exercise. First-time participants of HIIT may experience some fatigue after the exercise. The entire process will be accompanied by qualified first aid personnel and sports professionals. At the same time, participants in the intervention group will receive safety knowledge training to ensure that they can complete the exercise safely at home. Participants can stop immediately and inform the researchers if they experience any physical or mental discomfort during exercise.

The PI is responsible for ensuring that all research participants give informed consent before enrolling into the research.

Do you request a waiver of informed consent?

- ○ Yes
- ◉ No

Please upload Participant Information Sheet or Consent Form. Consent Form (15).docx (0.02MB)

Please upload Assent Form (if applicable).

Remuneration to Participant

Participant will be

- ○ Reimbursed, HKD
- ○ Not Reimbursed
- ◉ Others, please specify: Participants who successfully complete the research will receive sports products worth HK$400

- ◉ No

Do you have any potential conflicts of financial interest to declare? ○ Yes, please specify:

Data Storage and Access

Where will the data be stored?

All the data collected will be stored in a personal computer with a password of the investigator only.

Who will have access to the data?

Only the investigator can have access to the data.

What will happen to the data after research is completed?

All the data will be erased from the computer once the research is completed.

Will you collect data on audiotape, film/video, or other electronic media? No

Deception

Does your research involve the use of deception? No

Debriefing

Participants must be fully debriefed about the purpose of the research at the end of the study. A written debriefing handout must be provided to the participants.

Debriefing handout DEBRIEFING FORM (6).docx (0.02MB)

Declaration

Declaration ☑ I confirm that the information above is to the best of my knowledge accurate. I shall take reasonable care to ensure that the project is conducted in accordance with the Guidelines for Survey and Behavioural Research Ethics. I will obtain approval from other responsible units within CUHK (e.g. The Joint Chinese University of Hong Kong (CUHK) Hospital Authority New Territories East Cluster (NTEC) Clinical Research Ethics Committee (CREC), Animal Experimentation Ethics Committee (AEEC), University Safety Office/University Laboratory Safety Office) where appropriate.

Reviewer Assessment Checklist

☑ 1 ☑ 2 Risks to participants are minimized; research design does not unnecessarily expose subjects to risk.

☑ 1 ☑ 2 Risks to participants are reasonable in relation to anticipated benefits, if any, to subjects, and the importance of the knowledge that may reasonably be expected to result.

☑ 1 ☑ 2 Selection of subjects is equitable.

☑ 1 ☑ 2 Informed consent will be sought (or can be waived) from each prospective subject or the subject's legally authorized representative, and informed consent will be appropriately documented.

☑ 1 ☑ 2 There are adequate provisions to protect the privacy of participants and to maintain the confidentiality of data.

Status Log

Status	On	By (Role)	Remarks
Application Approved	2025-07-25 17:26	SBREC Final Approver	

SBREC Sec Validated	2025-07-22 10:26	Secretary of SBREC	
Approved by Second Reviewer	2025-07-21 10:08	2nd Reviewer	The risks have been well-considered and will be adequately managed by the PI.
Escalated to Second Reviewer	2025-07-21 09:33	1st Reviewer	
Under Review	2025-07-21 09:25	Secretary of Faculty Sub-committee	
Under Vetting	2025-07-21 09:25	Secretary of Faculty Sub-committee	
Submitted for Review	2025-07-20 11:24	Supervisor	
Reviewing by Supervisor	2025-07-19 15:34	Applicant	Regarding the concern from 2nd reviewer, the entire process will be accompanied by qualified first aid personnel and sports professionals. At the same time, participants in the intervention group will receive safety knowledge training to ensure that they can complete the exercise safely at home. Participants can stop immediately and inform the researchers if they experience any physical or mental discomfort during exercise. The revision can be seen in the Risk Description part.
Return for Revision	2025-07-17 09:07	Secretary of Faculty Sub-committee	Would PI please find below comment from the 2nd reviewer for follow up. Thank you.
Submitted for Review	2025-07-17 07:57	2nd Reviewer	I wish to know if personnel trained in first aid will be present at all times when participants are performing the exercises.
Escalated to Second Reviewer	2025-07-16 18:12	1st Reviewer	While I approve this protocol, it is better to have another colleague to take a look, given the demanding nature of the experiment.
Under Review	2025-07-16 10:47	Secretary of Faculty Sub-committee	
Under Vetting	2025-07-16 10:46	Secretary of Faculty Sub-committee	
Submitted for Review	2025-07-15 15:49	Supervisor	
Reviewing by Supervisor	2025-07-13 17:35	Applicant	
Draft	2025-07-13 16:23	Applicant	
